# fMRI mapping of the visual system in the mouse brain with interleaved snapshot GE-EPI

**DOI:** 10.1016/j.neuroimage.2016.06.015

**Published:** 2016-10-01

**Authors:** Arun Niranjan, Isabel N. Christie, Samuel G. Solomon, Jack A. Wells, Mark F. Lythgoe

**Affiliations:** a UCL Centre for Advanced Biomedical Imaging, Division of Medicine and Institute of Child Health, University College London, London, UK; b Centre for Cardiovascular and Metabolic Neuroscience, Department of Neuroscience, Physiology & Pharmacology, University College London, London, UK; c Department of Experimental Psychology, University College London, London, UK

**Keywords:** Mouse, fMRI, Negative BOLD, Visual cortex

## Abstract

The use of functional magnetic resonance imaging (fMRI) in mice is increasingly prevalent, providing a means to non-invasively characterise functional abnormalities associated with genetic models of human diseases. The predominant stimulus used in task-based fMRI in the mouse is electrical stimulation of the paw. Task-based fMRI in mice using visual stimuli remains underexplored, despite visual stimuli being common in human fMRI studies. In this study, we map the mouse brain visual system with BOLD measurements at 9.4 T using flashing light stimuli with medetomidine anaesthesia. BOLD responses were observed in the lateral geniculate nucleus, the superior colliculus and the primary visual area of the cortex, and were modulated by the flashing frequency, diffuse vs focussed light and stimulus context. Negative BOLD responses were measured in the visual cortex at 10 Hz flashing frequency; but turned positive below 5 Hz. In addition, the use of interleaved snapshot GE-EPI improved fMRI image quality without diminishing the temporal contrast-noise-ratio. Taken together, this work demonstrates a novel methodological protocol in which the mouse brain visual system can be non-invasively investigated using BOLD fMRI.

## Highlights

• Mouse brain fMRI data are improved using interleaved snapshot GE-EPI.
• Flashing light is a viable stimulus for task-based fMRI in the mouse.
• The lateral geniculate nucleus, superior colliculus and primary area of the visual cortex show reproducible BOLD responses.
• BOLD responses can be modulated with respect to flashing frequency.
• At a 10 Hz flashing frequency, negative BOLD responses are measured in the primary area of the visual cortex.

## Introduction

Understanding visual processing is a fundamental objective within neuroscience (Huberman and Niell, 2011) and the use of transgenic mouse models allows genetic influences on vision to be selectively investigated. The mouse brain visual system has been extensively studied by electrophysiological recordings (Grubb and Thompson, 2003, Niell and Stryker, 2008, Wang et al., 2010) and multi-photon microscopy (Kerr and Denk, 2008). These techniques provide a more direct measure of neuronal activity in comparison to the blood oxygenation level dependent (BOLD) signal typically measured in functional magnetic resonance imaging (fMRI) studies of human visual pathways. However, these methods and other optical techniques (Lim et al., 2015, White et al., 2011) require surgery to expose the brain, and can only measure limited sections of the brain at a time. Optical techniques are also generally limited to measurements of activity in accessible areas of the visual cortex. With fMRI, whole brain measurements can be made non-invasively and results compared more directly with human studies (Martin, 2014).

The application of fMRI to understand brain function in mice is growing. Efforts have been made to characterise many task-based (Adamczak et al., 2010, Ahrens and Dubowitz, 2001, Baltes et al., 2011, Bosshard et al., 2010, Nair and Duong, 2004, Schroeter et al., 2014) and resting state functional systems (Guilfoyle et al., 2013, Jonckers et al., 2011, Nasrallah et al., 2014, Sforazzini et al., 2014, Stafford et al., 2014). Surprisingly, however, there is only a single study investigating the mouse brain visual system using fMRI (Huang et al., 1996). That study reported highly atypical BOLD responses when considered against rat brain data (Bailey et al., 2013, Lau et al., 2011a, Lau et al., 2011b, Pawela et al., 2008, Van Camp et al., 2006).

fMRI is technically challenging in the mouse in comparison to larger mammals. The smaller size of the mouse brain amplifies many of the issues faced in fMRI, where the goal is to maintain sufficient sensitivity to BOLD signal changes at reasonable spatial and temporal resolution. A single-shot GE-EPI pulse sequence is the standard method of acquiring fMRI data. However single-shot GE-EPI is vulnerable to local field magnetic gradients caused by bulk magnetic susceptibility discontinuities and inhomogeneities, resulting in signal loss and image distortion. This is particularly apparent in the mouse brain, due to the low volume/surface area ratio (Adamczak et al., 2010). Interleaved snapshot GE-EPI (Guilfoyle and Hrabe, 2006) has been suggested as an alternative acquisition protocol that can reduce susceptibility induced artefacts without compromising temporal resolution. Briefly, the conventional EPI sequence is separated into a series of excitation/acquisition snapshots conducted in succession at varied flip angles within one TR period. Each snapshot partially fills k-space (Fourier image space) and the entirety of k-space is composed of the interleaved snapshots. Each slice is acquired in turn with *n* snapshots, reducing vulnerability to respiration artefacts faced by conventional segmented EPI sequences.

Guilfoyle et al. showed an improvement in spatial localisation of the BOLD signal with increasing *n*, accompanied by a reduction in image signal-to-noise ratio (SNR) proportional to$\;\sqrt{n}$. However, it is unclear how a reduction in image SNR impacts in the temporal domain, particularly with regards to the contrast-to-noise (CNR), which is the most useful quality metric in fMRI. Despite the potential benefits, snapshot GE-EPI has yet to be applied to task-based fMRI of the rodent brain, and so the first part of this study evaluated this technique with application to mouse brain fMRI. We hypothesised that by increasing *n*, marked improvements in spatial localisation of the BOLD signal would be observed but at cost to image SNR, temporal SNR and CNR (that may be an acceptable penalty for many future applications given the marked image distortion previously reported (Adamczak et al., 2010)).

In the second part of this study we presented flashing visual stimuli to both eyes against a dark background, to evoke BOLD signals and characterise the functional response of the mouse brain visual system with stimulus modulation. We focus on three brain regions (defined in the Allen Mouse Brain Atlas (Lein et al., 2007)) of particular importance in visual processing: the dorsal lateral geniculate nucleus(LGd) of the thalamus, the sensory layers of the superior colliculus (SCs) and the primary visual area of the cortex (VISp) otherwise known as V1 (Huberman and Niell, 2011). Based on previous fMRI experiments conducted in the rat (Bailey et al., 2013, Pawela et al., 2008), we examined the dependence of the BOLD response on the flashing frequency *f* of the stimulus (in range 1–10 Hz). We hypothesised positive BOLD responses in all three regions for all *f*. In addition, we expected positive trends in BOLD response magnitude with *f* in the LGd and SCs, and a negative trend in VISp.

For the final part of the study we aimed to demonstrate the sensitivity of our method to detect differential patterns of BOLD activation driven by specific visual stimuli. Existing work suggests that most cells in the SCs respond to both ‘on’ or ‘off’ stimuli (Wang et al., 2010). What is not clear is the relative strength of responses to large ‘on’ or ‘off’ stimuli across the neuronal population in the SCs. Indeed, the most numerous cell-type of the mouse retina, shows stronger ‘off’ than ‘on’ responses (Zhang et al., 2012), and ‘off’ stimuli are thought to be particularly effective in both driving innate behavioural responses in mice (Yilmaz and Meister, 2013) and driving neurons in the mouse superior colliculus (Zhao et al., 2014). We therefore hypothesised that the SCs region is preferentially responsive to dark flashes against a bright background as opposed to light flashes against a dark background, and that dark flashes would therefore elicit stronger BOLD responses in the SCs. In addition, we used monocular stimulation, and hypothesised stronger BOLD responses in the contralateral hemisphere for the LGd, SCs and VISp, in accordance with the dominance of contralateral retinal projections.

The technical issues inherent to fMRI imaging protocols are further compounded by the relative difficulty of monitoring and maintaining normal animal physiology (crucial for robust fMRI signals in the anaesthetised rodent brain). In this study we use a minimally invasive, free-breathing protocol with medetomidine anaesthesia at recoverable doses, as previously described in the literature (Adamczak et al., 2010, Jonckers et al., 2011, Nasrallah et al., 2014). Such a strategy may be advantageous for future studies that aim to investigate longitudinal functional changes in the same cohort of mice, and for high-throughput screening studies.

## Materials and methods

### Animals

All experiments were performed in mice in accordance with the European Commission Directive 86/609/EEC (European Convention for the Protection of Vertebrate Animals used for Experimental and Other Scientific Purposes) and the United Kingdom Home Office (Scientific Procedures) Act (1986) with project approval from the Institutional Animal Care and Use Committee. All mice were acclimatised two weeks prior to data acquisition in an animal house maintained at a temperature of 21 ± 2 °C and a relative humidity of 55 ± 10%, on a 12 hours light/12 h dark cycle with a 30 min twilight switch.

26 female C57BL6/J mice weighing 20.5 ± 1.0 g were used in total. 6 were used to investigate the use of snapshot GE-EPI in mouse brain fMRI (experiment 1), 8 to characterise the BOLD functional response of the visual pathway to stimulus frequency (experiment 2), and 12 to study the effect of flash context (a bright background with dark flashes vs a dark background with bright flashes) (experiment 3). Anaesthesia was induced with isoflurane gas (2%) and maintained with medetomidine (0.4 mg/kg bolus, 0.8 mg/kg infusion initiated 10 min after bolus) (Adamczak et al., 2010) administered subcutaneously. A gas mixture of 0.1 L/min of O_2_ and 0.4 L/min of medical air (BOC Healthcare (Linde AG), Munich, 20.9 ± 0.5% O_2_ with balance composed of N_2_) was continuously supplied via a nose cone during imaging. Following administration of the medetomidine bolus, isoflurane was gradually discontinued at a rate of 0.2% per minute. This anaesthetic regime produced a stable respiratory rate of 159 ± 25 breaths per minute. Eye gel was used to prevent drying of the corneas, and ear bars were used with analgesic cream to minimise head motion. Core body temperature was maintained at 37.1 ± 0.3 °C.

Respiratory rate was measured using a pressure sensitive pad, and core body temperature was measured using a rectal thermometer (SA Instruments). Core body temperature was maintained using a warm water circuit and hot air fan feedback system (SA Instruments). Previous bench experiments measured mean arterial O_2_ saturation to be 97.6% (data not shown) under these conditions using a MouseOx pulse oximeter (Starr Life Sciences), in line with similar results in the literature (Nasrallah et al., 2014).

### MRI methods

All MRI experiments were performed on a 9.4 T VNMRS horizontal bore MRI scanner (Agilent Inc., Palo Alto, CA) with an Agilent 205/120HD gradient set. For experiments 1 and 2, a 72 mm inner diameter volume coil for RF transmission (Rapid Biomedical), and a room-temperature 2 channel array surface coil (Rapid Biomedical) for signal reception. For experiment 3, a custom-built single loop surface coil was used for both RF transmission and reception. VNMRJ 3.1 software was used for image acquisition and reconstruction.

An anatomical reference scan was taken using a Fast Spin Echo sequence (TR/TE_eff_ = 4000/48 ms, ETL = 8, matrix size = 192 × 192, FOV = 35 × 35 mm^2^, 35 coronal slices each 0.6 mm thick). fMRI data were acquired using GE-EPI (FOV = 35 × 35 mm^2^, matrix size = 96 × 96, 12 coronal slices each 0.5 mm thick, slice gap 0.1 mm, spectral width = 178.6 kHz, TR = 2.5 s, TE = 19 ms, one EPI triple reference image). The acquisition time per snapshot, T_seg_, was set to 50.18 ms for all sequences. Experiments 1 and 2 used 84 volumes, whilst experiment 3 used 131 volumes. The anatomical reference scan ensured whole brain coverage, and the fMRI slices were positioned anterior to the anterior aspect of the cerebellum (Huang et al., 1996). Shimming was conducted using a GE 3D protocol (Vanzijl et al., 1994, Webb and Macovski, 1991), with both 1st and 2nd order shims optimised in a user defined shim voxel (approximately 5 × 8 × 9 mm^3^) with voxel edges set at the brain edge. Typical line-width (FWHM) within this shim voxel was approximately 60 Hz.

In experiment 1, fMRI data were collected using a number of GE-EPI snapshots *n* ranging from one to four. At each *n*, the required flip-angle for each shot was calculated according to methods described elsewhere (Guilfoyle and Hrabe, 2006). For single-shot scans, k-space was under-sampled with 48 rows collected per slice and then zero-filled to 96, to maintain a constant echo time given the limitations of the gradient hardware. After investigating the effect of *n* on CNR, fMRI data were acquired using *n* = 4 for experiments 2 and 3.

### Visual stimulation

Stimulation timings were triggered from the beginning of the EPI sequence using a POWER1401 control system (CED Ltd., UK) with Spike2 software. For experiments 1 and 2, the stimulus consisted of blue laser light (445 nm, Omicron) transmitted into the scanner bore using a fibre optic cable. The cable was placed dorsal to the mouse head, secured to the top of the surface coil and aimed into the bore; in order that light reflected off the surface of the coil interior. This way, the eyes could be stimulated bilaterally with diffuse light without risk of retinal damage. When investigating the effect of *n* on CNR, the laser was pulsed at a frequency of 10 Hz, with pulse duration of 10 ms, and a laser current of 10 mA. The output power was measured to be 0.72 mW at the end of the fibre optic cable. During baseline periods the laser power output was zero. When investigating the BOLD response dependence on *f*, frequencies of 1, 3, 5 and 10 Hz were chosen based on previous studies of the rat brain (Bailey et al., 2013, Pawela et al., 2008, Van Camp et al., 2006).

The stimulus was delivered using a block design paradigm of 40 s rest, 20 s activation alternately repeated three times. Each fMRI scan was conducted twice at each condition (either *n* or *f*) in a pseudo-random order. The 8 fMRI scans were acquired over 40 mins for each subject. This resulted in 6 activation periods per condition per subject.

For experiment 3, a cold white LED (Thor Labs) was used in conjunction with a custom-built eye-piece attached to the fibre optic cable for monocular stimulation. In order to use this eye-piece to deliver visual stimuli whilst acquiring fMRI data, it was necessary to use a different MRI coil set-up to allow space for the eye-piece and avoid placing undue stress on the mouse head. Two conditions were tested. Condition 1 used a dim but non-zero baseline intensity (20 mA) with bright flashes (1000 mA) with dark intervals (0 mA). Condition 2 used a bright baseline (980 mA) with dark flashes (0 mA) with bright intervals (1000 mA). The output power at the end of the fibre optic cable with the eye-piece for input current of 1000 mA was measured to be 0.15 mW. Pulse duration was 10 ms, and a 2 Hz pulse flashing frequency used during periods of activation. Both conditions used a block design of 40 s rest, 20 s activation alternately repeated five times. Each fMRI scan was conducted twice for each condition, resulting in 10 activation periods per condition per subject.

### Data analysis

All data analysis was conducted using ITK-SNAP (Yushkevich et al., 2006), NiftiReg (Ourselin et al., 2000), in-house MATLAB 2014b scripts, SPM12 (Penny et al., 2011), SnPM13 (Nichols and Holmes, 2002), the MarsBaR toolbox (Brett et al., 2002) and GraphPad Prism 6. All voxel size information was increased in size by a factor of ten to facilitate the use of SPM (originally designed for use with human sized brains), however all distances and locations are reported in real space. Anatomical reference scans were registered to the reference scan (manually skull-stripped using ITK-SNAP) of the final subject of each experiment using an affine registration with NiftiReg, and the affine transformation matrix generated was then applied to the fMRI data. To generate structural ROIs, the Allen histology mouse brain atlas (Lein et al., 2007) was directly registered to the data in the same way, and atlas labels transformed accordingly. The registration was evaluated by visual inspection with respect to the anatomical reference scan using SPM12 and the Paxinos Mouse Brain Atlas (Paxinos and Franklin, 2004). After registration the fMRI data were realigned, corrected for differences in slice timing and smoothed (Gaussian FWHM of two voxels). The first image was discarded before slice timing correction.

Region-of-interest (ROI) analysis was conducted by using atlas labels to extract timecourses using MarsBaR, in a bid to avoid circularity (Kriegeskorte et al., 2009). The labels chosen for timecourse extraction were the LGd, SCs and VISp, which correspond to the dorsal lateral geniculate nucleus, the sensory areas of the superior colliculus and the primary visual area, commonly referred to V1. Where the stimulus was binocular in nature, ROIs included both brain hemispheres. As experiment 3 used monocular stimulation, labels were sub-divided by hemisphere. The MarsBaR source code was altered in order that individual voxel timecourses were filtered and normalised before averaging. Timecourses were normalised to percentage signal change by dividing each value by the mean value of the whole timecourse. BOLD contrast was then calculated by subtracting the mean preceding baseline value from the mean BOLD value from each stimulus epoch. Temporal CNR was calculated by dividing the mean BOLD contrast by the standard deviation of the BOLD signal in the baseline period (Welvaert and Rosseel, 2013). Image SNR was calculated by dividing the mean intensity of the first time point in the SCs by the standard deviation of an equivalent sized ROI centred outside the brain.

For experiment 1, linear regression was performed on BOLD temporal CNR values and temporal SNR in the SCs to test for trends with respect to *n*, and on image SNR with respect to $\;\sqrt{n}$. For experiment 2, linear regression was performed on the BOLD contrast values in the LGd, SCs and VISp to test for trends with respect to *f*. For experiment 3, two-way ANOVA was performed on BOLD contrast values in the LGd, SCs and VISp to test for differences between the two conditions, with ipsi/contra-lateral hemisphere and stimulus condition set as independent factors. Where interactions were not significant at the 5% level, main effects were reported. Post-hoc two-tailed paired *t*-tests were then performed where factor interactions were significant at the 5% level, in order to report simple main effects (Maxwell and Delaney, 2004).

For statistical parametric mapping, 1st-level general linear model (GLM) analysis was conducted for each subject under each condition, with both fMRI scans included in the GLM with estimated motion parameters as nuisance regressors (single subject fixed effects model). Voxels were only analysed if they were included in a brain mask manually generated from the anatomical reference scan of the last subject. The SPM12 canonical HRF was convolved with the stimulus profile as the explanatory model. The default SPM12 options of grand mean scaling and auto-correlation modelling were used, with a high-pass filter of 128 s. For experiments 1 and 2, a two-tailed *t*-test was then performed on a voxel by voxel basis to test the null hypothesis that the BOLD signal is not explained by the explanatory model. Based on the results from experiment 2, a one-tailed *t*-test was used for experiment 3. All statistical parametric maps shown were corrected for multiple comparisons using a FWER (p < 0.05) threshold determined by random field theory using SPM12 unless otherwise stated. No cluster thresholding was used.

To understand group level activations, both fixed (FFX) and mixed (RFX) effects analyses were conducted. The fixed effects group analysis included all subject scans for each condition in the same GLM (with appropriate nuisance regressors). The mixed effects group analysis included the contrast images outputted by the initial single subject fixed effects analyses. Due to the relatively low number of subjects used in this study, non-parametric methods (SnPM13) were used to define statistical significance thresholds for mixed effects analysis (see Supplementary material).

## Results

### Identification of visual system BOLD responses

**Fig. 1:**
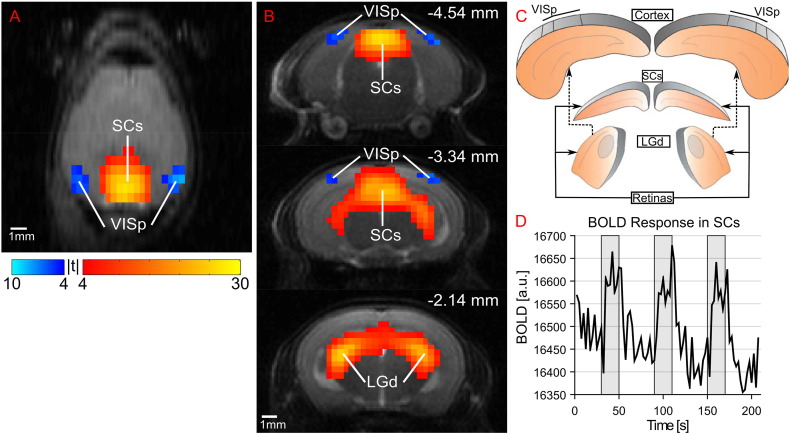
Fixed effects analysis (two-tailed *t*-test, FWE p < 0.05, N = 6) statistical parametric map generated from snapshot GE-EPI (N = 4), overlaid on anatomical reference image in A) transverse view and B) three coronal slices (with distance relative to Bregma). C) Schematic of mouse brain visual system (minus the chiasma), adapted with permission (Huberman and Niell, 2011). D) Representative mean BOLD timecourse in the SCs from a single session. Stimulus epochs are shown by grey regions, and in these time periods the BOLD signal increases by approximately 1%.

Bilateral BOLD responses to a flashing light visual stimulus were identified in the LGd, SCs and VISp regions through fixed effects SPM analysis. Results from the 10 Hz stimulus measured with GE-EPI using *n* = 4 snapshots are shown in Fig. 1.

Regions of BOLD activation that show close spatial affinity to the mouse visual pathway were observed (Fig. 1A–C). The spreading of the BOLD response beyond these regions in a ‘halo’ effect is likely due to the 2-voxel FWHM smoothing kernel applied in the pre-processing step, as recommended by (Poldrack et al., 2011). An alternative explanation is the presence of draining veins surrounding the thalamus, which has been noted previously in gradient echo BOLD imaging (Lee et al., 1999). Fig. 1D shows the mean BOLD timecourse measured in the SCs from a single animal over the course of a single scan with three stimulus epochs. At 10 Hz, clear bilateral negative BOLD responses can be seen in VISp, with positive BOLD responses in the SCs and LGd.

### Effect of varying GE-EPI snapshot number on CNR and image distortion (experiment 1)

**Fig. 2:**
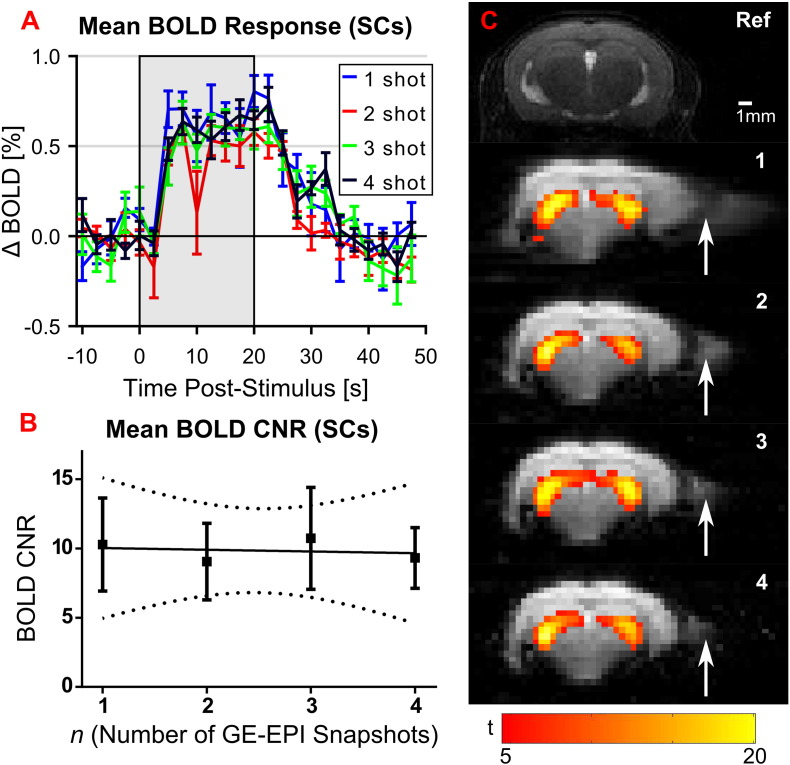
Effects of using snapshot GE-EPI for mouse brain fMRI. A) Mean BOLD response in SC measured with increasing number of GE-EPI snapshots (± S.E.M) (N = 6). B) Mean BOLD CNR in SCs (± S.E.M) (N = 6). C) Representative GE-EPI from single subject showing reduction in distortion (white arrow) with increasing snapshot number, with anatomical reference image (Ref). Single subject fixed effects statistical map (FWE p > 0.05) is overlaid for each snapshot number, showing activation patterns in the LGd.

To examine CNR when using interleaved snapshots, the peak BOLD timecourse intensity in each stimulation epoch was divided by the standard deviation of BOLD signal during 15 s of the baseline period directly preceding it. Mean BOLD responses to the visual stimulus and temporal CNR measurements in the SCs are shown in Fig. 2.

No loss in temporal CNR with increasing snapshot number was observed (Fig. 2A and B). Linear regression showed no trend in BOLD CNR values across snapshots (p = 0.9259). Temporal SNR in the baseline period of the SCs also exhibited no trend with *n* (p = 0.9044). As expected, a decrease in image SNR with the square root of *n* was seen (p = 0.0065). Importantly, image distortion was markedly reduced, and the symmetry of BOLD activation was noted to increase with increasing snapshot number (Fig. 2C). Signal dropout towards the base of the brain did not appear to be affected by snapshot number. A summary of the quality assurance measures is shown in Table 1.

### BOLD response dependence on stimulus frequency (experiment 2)

**Fig. 3:**
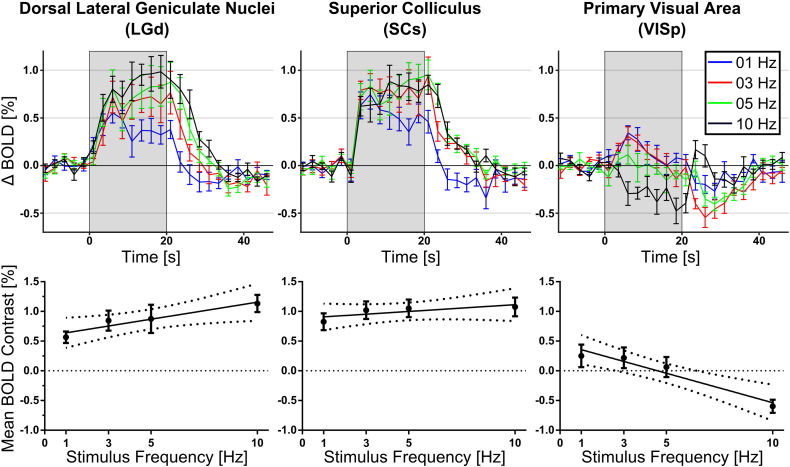
BOLD responses and contrasts in the LGd, SCs and VISp regions. BOLD timecourses (top) are plotted as means ± S.E.M. (N = 8). Trends in mean BOLD contrast (bottom) are plotted with 95% confidence intervals.

After consistently observing negative BOLD responses in VISp at 10 Hz in experiment 1, fMRI was performed in a cohort of mice (N = 8) with variable stimulus flashing frequency (1–10 Hz). Mean BOLD responses to the visual stimulus at different frequencies and corresponding mean peak BOLD contrasts for the LGd, SCs and VISp are shown in Fig. 3.

Positive trends in BOLD contrast with frequency were seen in both the LGd and SCs, and a negative trend found in VISp. The negative BOLD response at 10 Hz in the VISp was found in both cohorts of animals used in experiments 1 and 2. Results for experiment 2 are summarised in Table 2, and show that over the range of frequencies tested, VISp has a stronger frequency preference than sub-cortical regions.

### BOLD response dependence on background intensity level with monocular stimulation (experiment 3)

**Fig. 4:**
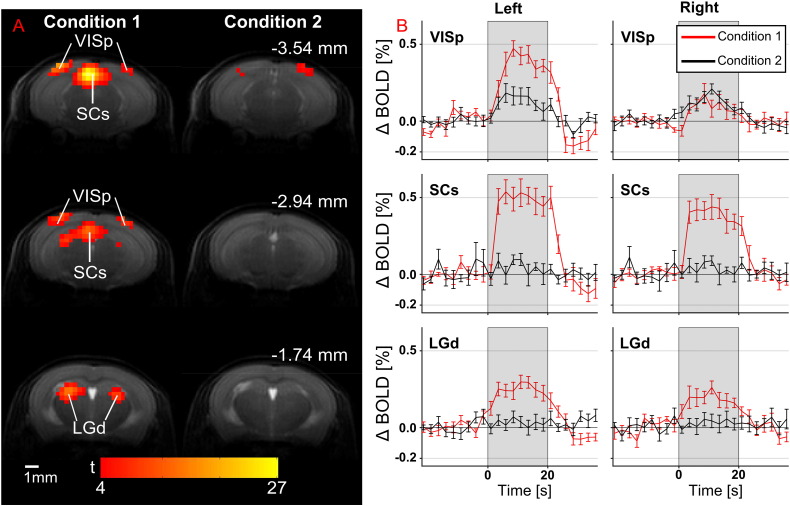
BOLD responses to monocular stimulation of the right eye with white light at 2 Hz flashing frequency using bright flashes (condition 1) and dark flashes (condition 2). A) FFX statistical parametric maps overlaid on an anatomical reference scan (one-tailed *t*-test, FWE p < 0.05), for three coronal slices (distances measured from Bregma). BOLD responses appear stronger in the contralateral hemisphere. B) BOLD percentage change against time for left and right VISp, SCs and LGd. Bright flashes against a dark background elicit stronger BOLD responses than dark flashes against a bright background.

The BOLD response to monocular stimulation was measured, and the BOLD responses to bright flashes against a dark background (condition 1) against dark flashes against a bright background (condition 2) were investigated in a separate cohort of 12 subjects using a cold white LED light source and a custom-built eye-piece. Mean BOLD responses in both hemispheres for the LGd, SCs and VISp are shown in Fig. 4.

A two-way ANOVA was performed on BOLD contrasts for the LGd, SCs and VISp regions, with condition and hemisphere as factors (both repeated measures). These results are summarised in Table 3.

The interaction between hemisphere and condition is significant at the 5% level in both the SCs and VISp regions. In the LGd this interaction is not significant, and therefore it is reasonable to directly report a significant main effect of condition on BOLD contrast, but not hemisphere. As the interaction term was found to be significant in both the SCs and VISp, simple main effects for condition and hemisphere are reported in Table 4.

By thresholding at α = 0.05, the results in Table 4 suggest there are significant differences in the following pairwise comparisons: between hemispheres during condition 1 in both the SCs and VISp; between conditions in the SCs and VISp in the contralateral hemisphere; between conditions in the ipsilateral SCs.

## Discussion

This study aimed to investigate the use of GE-EPI with interleaved snapshots for mouse brain fMRI and characterise the BOLD functional response of the mouse brain to visual stimuli. An improvement in fMRI images with a greater number of snapshots was shown, without a reduction in temporal CNR. Robust BOLD responses were recorded in the mouse visual system, including negative BOLD responses (NBRs) in the VISp region at 10 Hz stimulus frequency. Monocular stimulation evoked stronger BOLD responses on the contralateral hemisphere as expected. Dark flashes against a bright background elicited weak BOLD responses in VISp without responses in the LGd or SCs, whereas bright flashes against a dark background at 2 Hz induced positive BOLD responses (PBRs) across the visual pathway.

### The use of GE-EPI with interleaved snapshots

The improvement in spatial localisation of the BOLD signal by using multiple snapshots has already been documented in mouse brain MRI (Guilfoyle and Hrabe, 2006). Theory predicts that as *n* increases, image SNR should decrease by a factor of$\;\sqrt{n}$. Data from experiment 1 confirms this reduction, but shows no appreciable detriment to temporal CNR (over the range *n* [1–4]). The link between image SNR and temporal CNR is non-trivial, as both hardware and physiology contribute to noise in fMRI. Under the current experimental conditions, this finding indicates that physiological noise dominates hardware noise in the temporal domain. Equally, this suggests that snapshot GE-EPI represents a highly advantageous approach to reduce image distortion in GE-EPI data with no fMRI sensitivity cost, and should be considered in future studies.

There are alternatives to interleaved snapshot EPI for mouse brain fMRI, such as conventional segmented EPI or parallel imaging using multiple coils. Conventional segmented EPI sequences are more susceptible to motion artefacts, as there is a longer time between segment acquisitions in the same slice. Parallel imaging is commonly used in human fMRI, as it collects all data segments simultaneously. However this is highly dependent on coil geometry and benefits most from large coil arrays. The small size of the mouse brain makes parallel imaging less suitable than interleaved snapshot GE-EPI (Guilfoyle and Hrabe, 2006).

### Visual stimulation

In experiments 1 and 2, visually evoked BOLD responses were measured in response to a flashing light visible to both eyes. Only one previous study has used fMRI to study the mouse visual system (Huang et al., 1996), and our work builds on this by using GLM analysis to map BOLD activation and unbiased structural ROIs to extract BOLD time series data. Our analyses show BOLD activation in regions known to be part of the visual pathway, and we observed statistically significant linear trends in the BOLD response at different temporal frequencies, in both LGd and VISp. Binocular stimulation induced symmetric BOLD responses across the visual system (N = 6, N = 8). By contrast, monocular stimulation (experiment 3; N = 12) induced spatially larger BOLD responses in the contralateral hemisphere than in the ipsilateral hemisphere. This is expected because the central projections of the mouse retina show contralateral bias.

The long, narrow scanner bores and high field strengths of pre-clinical MRI systems, in addition to susceptibility to radio frequency interference, make it more difficult to conduct the complex visual tasks that are possible using techniques such as electrophysiological recordings and multi-photon microscopy. Future experiments may improve on the visual stimulation used here by using an array of fibre optic cables to convey spatially-varying images into the bore, as demonstrated by fMRI experiments in the rat brain (Lau et al., 2011a). Such an approach could provide comparative fMRI data that complements the existing literature of electrophysiology studies in mouse visual pathways that have used spatially structured visual stimuli to explore visual responses in cortex (Niell and Stryker, 2008), SCs (Wang et al., 2010) and LGd (Grubb and Thompson, 2003).

### Frequency dependence of the BOLD response

In experiment 1 (N = 6), the use of a blue light source flashing at 10 Hz elicited PBRs in the mid-brain, and NBRs in VISp. Experiment 2 (N = 8) examined the dependence of the BOLD response on temporal frequency, and demonstrated PBRs in the VISp at lower frequencies. The frequency-dependence of PBRs in the mid-brain concurs with similar studies conducted in the rat brain (Bailey et al., 2013, Pawela et al., 2008, Van Camp et al., 2006). NBRs have not been reported previously, however a trend for a reduced amplitude of evoked potentials with increasing stimulus frequency has been previously observed in the rat visual cortex (Pawela et al., 2008), in concordance with the observed trends in BOLD signal with frequency in the present work.

Whilst NBRs have not been directly measured in the mouse brain before, they were predicted to arise in mice as a consequence of anaesthesia interference in neurovascular coupling (Sharp et al., 2015). That study, using a novel anaesthesia protocol and measuring responses of somatosensory cortex, showed abnormal responses of both blood vessels and haemoglobin compounds during whisker stimulation. These abnormal responses predict an inverted BOLD response, the temporal profile of which corresponds closely to the NBRs we have measured. The abnormal responses returned to normal 4 h post-anaesthesia induction, which led Sharp et al. to conclude that anaesthesia was the primary cause of any breakdown in neurovascular coupling that might lead to NBRs. The data in the present work demonstrates selective induction of NBRs not by variation in anaesthesia but by a stimulus characteristic (temporal frequency), which may represent a useful method for future in-vivo study of this phenomenon.

One possible explanation for the NBRs observed in this work is a non-linear neurovascular coupling: that at higher temporal frequencies neurovascular coupling is itself compromised, which has previously been suggested in rat visual fMRI/electrophysiology data (Bailey et al., 2013). An alternative explanation assuming linear behaviour in neurovascular coupling is that at higher frequencies, inhibitory neurons elsewhere in the visual system reduce neuronal activity in VISp, which in turn trigger NBRs. This explanation is supported by fMRI studies in macaque monkeys (Shmuel et al., 2006) and humans (Chen et al., 2005, Shmuel et al., 2002, Tootell et al., 1998). However the present data cannot differentiate between these two hypotheses.

### Background context of light flashes

Experiment 3 was conducted to determine if our protocol could detect other, potentially subtle stimulus-dependent changes in BOLD responses. The initial hypothesis was that the SCs would respond preferentially to dark flashes (condition 2) relative to light flashes (condition 1). The data suggests the opposite – with condition 1 eliciting similar BOLD responses seen in data from experiments 1 and 2, and condition 2 only inducing appreciable BOLD increases in VISp. The difference in BOLD responses across conditions is marked, and statistically significant effects at the 5% level were seen for both hemisphere and condition factors across the visual pathway. Monocular stimulation produced hemispheric differences in the BOLD response in VISp and SCs, but not in LGd. This appears consistent with the topography of these regions: in VISp and SCs, contra- and ipsilateral inputs are generally segregated with limited binocular overlap, whereas in LGd contralateral inputs approximately encase ipsilateral ones, in both hemispheres. At the resolution of our scans, voxel size would not be small enough to resolve topography of LGd, and may mean that hemispheric difference in neuronal activity in LGd are not reflected by changes in BOLD signal. The greater overall responses to light flashes on a dim background, than dark flashes on a bright background, may reflect differences in the adaptation state of the retina. That responses to dark flashes are stronger in visual cortex may suggest that the visual cortex is more closely associated with the interpretation of dark temporal edges, relative to subcortical regions.

### Animal physiology

There are two general strategies to obtaining fMRI measurements from anaesthetised mice. One option is to use neuromuscular blocking agents with mechanical ventilation, which allows control of respiratory rate/volume and blood gas levels, and minimises head motion (Baltes et al., 2011, Bosshard et al., 2010, Grandjean et al., 2014, Schroeter et al., 2014). However, mechanical ventilation via cannulation of the trachea is invasive, whilst endotracheal intubation is technically challenging in the mouse. The second option, as done here, is to use a free breathing protocol (Adamczak et al., 2010, Nasrallah et al., 2014). This enables recovery, and thus longitudinal studies, but may increase the between-subject variability.

Anaesthesia effects on mouse fMRI responses are well documented for paw electrical stimulation at innocuous intensity levels, and a previous study recorded a 10 s lag between stimulus onset and BOLD response in the somatosensory cortex under medetomidine and urethane anaesthesia (Schroeter et al., 2014). We saw no such lag using a medetomidine only protocol, with a larger bolus and infusion concentration delivered subcutaneously as opposed to a tail vein injection. The lag effects are also not indicated in other paw stimulation studies (Adamczak et al., 2010, Nasrallah et al., 2014) in the mouse that used medetomidine, at an intermediate dose (0.3 mg/kg bolus, 0.6 mg/kg/h infusion). We have used higher does because in pilot studies we found that this dose was too low to robustly minimise hindpaw reflex in the C57BL/6 J mouse strain. Thus in this work a 0.4 mg/kg bolus, 0.8 mg/kg/h infusion of medetomidine was used, which minimised the hindpaw reflex whilst maintaining a robust BOLD response to visual stimulus.

## Conclusion

Mouse brain fMRI has been demonstrated using a bilateral visual stimulus to simultaneously map the LGd, SCs and VISp regions of the visual pathway. Data acquired were comparable to rat data in earlier studies (Bailey et al., 2013, Lau et al., 2011a, Lau et al., 2011b, Pawela et al., 2008, Van Camp et al., 2006), whilst considerably improving on the single mouse visual fMRI study reported in the literature (Huang et al., 1996). Using GE-EPI with up to four interleaved snapshots showed no reduction in temporal CNR, whilst reducing susceptibility induced image distortions. The dependence of BOLD response on stimulus temporal frequency was measured across the visual system, with negative BOLD responses elicited in VISp at 10 Hz, due either to a breakdown in neurovascular coupling or a reduction in neuronal activity. The preference of the visual pathway for bright flashes as opposed to dark flashes was clearly demonstrated.

In addition to mapping the visual pathway, the method presented in this work provides a practical solution for mouse brain fMRI. The method employs a free-breathing medetomidine anaesthetic protocol at recoverable doses, and does not require cryogenically-cooled surface receiver coils for robust data acquisition. This approach may be valuable for future studies that aim to investigate interactions between genetics and functional brain responses to visual stimuli using fMRI.

## Figures and Tables

**Table 1:** Linear regression results for experiment 1, testing for dependence of fMRI quality metrics on number of EPI snapshots *n*.

Metric	Model	*m*	95% Confidence interval for *m*	F (DFn = 1, DFd = 22)	p-Value
Image SNR	$y=m\sqrt{n}+c$	− 51.26 [ΔSNR n^-0.5^]	[− 86.63–15.90]	9.037	0.0065
Temporal SNR	*y* = *mn* + *c*	3.072 [ΔSNR n^− 1^]	[− 49.36 55.50]	0.01476	0.9044
Temporal CNR	*y* = *mn* + *c*	− 0.123 [ΔSNR n^− 1^]	[− 2.834 2.588]	0.00859	0.9259

**Table 2:** Linear regression results for experiment 2, testing for dependence of BOLD contrast on flashing frequency *f* in the visual system.

ROI	*m* [Δ% Hz^− 1^]	95% Confidence interval for *m*	F (DFn = 1, DFd = 30)	p-Value
LGd	0.0489	[0.00152 0.0963]	4.44	0.0436
SCs	0.0200	[− 0.0188 0.0588]	1.11	0.3010
VISp	− 0.0532	[− 0.0754–0.0312]	24.3	0.0000287

**Table 3:** Summary of two-way repeated measures ANOVA on BOLD contrasts in LGd, SCs and VISp regions, with stimulus condition and hemisphere as repeated factors.

ROI	Source of variation	F (DFn = 1, DFd = 11)	p-Value
LGd	Hemisphere factor	1.404	0.2611
Condition factor	28.43	0.0002
Interaction hemisphere × Condition	1.722	0.2161
SCs	Hemisphere factor	5.470	0.0393
Condition factor	33.88	0.0001
Interaction hemisphere × Condition	5.130	0.0447
VISp	Hemisphere factor	10.72	0.0074
Condition factor	3.441	0.0906
Interaction hemisphere × Condition	11.43	0.0061

**Table 4:** Simple main effects in the SCs and VISp, examined using post-hoc two-tailed paired *t*-tests (df = 11, no correction for multiple comparisons).

ROI	Factor	Post-hoc paired *t*-test	Mean difference C [Δ% BOLD Contrast]	95% Confidence interval for C	*t*-Statistic	p-Value
SCs	Contralateral	Condition 1 - Condition 2	0.4493	[0.2898 0.6088]	6.201	0.00007
Ipsilateral	Condition 1 - Condition 2	0.3648	[0.2057 0.5239]	5.046	0.00037
Condition 1	Contralateral - Ipsilateral	0.0941	[0.0088 0.1794]	2.428	0.03353
Condition 2	Contralateral - Ipsilateral	0.0095	[− 0.0198 0.0388]	0.717	0.48832
VISp	Contralateral	Condition 1 - Condition 2	0.2266	[0.0726 0.3806]	3.238	0.00790
Ipsilateral	Condition 1 - Condition 2	− 0.0360	[− 0.1643 0.0923]	0.618	0.54922
Condition 1	Contralateral - Ipsilateral	0.2678	[0.1205 0.4152]	4.000	0.00209
Condition 2	Contralateral - Ipsilateral	0.0053	[− 0.0934 0.1040]	0.117	0.90881

## Appendix A Supplementary data

Supplementary material. Image 1
