# Supplementary material for: fMRI mapping of the visual system in the mouse brain with interleaved snapshot GE-EPI
Source: Neuroimage. 2016 Oct 1;139:337–45. doi: 10.1016/j.neuroimage.2016.06.015 (PMC4988789; doi:10.1016/j.neuroimage.2016.06.015)
Supplement: Supplementary file 1 — Supplementary material. [file mmc1.docx]

**Generalising to populations**

The use of mixed effects GLM analysis is required to make statistical inferences describing the population from which the subjects are derived. This requires a higher statistical threshold to be passed than fixed effects analysis due to the fewer degrees of freedom of the t-distribution which is sampled. This is also a reflection of the larger power requirements for effects to be detected under mixed analysis. For group sizes of 6 and 8, mixed effect BOLD responses were undetectable at conventional statistical thresholds (FWER p < 0.05, |t| > 10.69, 5-7 degrees of freedom). However, both groups included subjects scanned with a 10 Hz frequency using GE-EPI with four snapshots. Mixed effects analysis (FWER p < 0.05, two-tailed t-test) was performed on this combined group (N = 14).

As the number of subjects was still relatively low, it was possible to use permutation methods to generate maximum t-statistic distributions for choosing appropriate thresholds. Whilst less common, this method makes fewer assumptions about the data, and performs a non-parametric permutation test at each and every voxel. There is therefore no longer a one-to-one mapping from t-statistic to p-value. The permutation testing was conducted using the results of the 1^st^ level statistical mapping using a variance smoothing kernel of twice the voxel size. The results of this analysis are shown in Figure S1. From this analysis, we can be confident that the PBRs measured in the LGd and SCs, and the NBRs measured in the VISp are generalizable to the population i.e. that the maps have predictive power for future experiments, as well as describing the data from which they are derived.


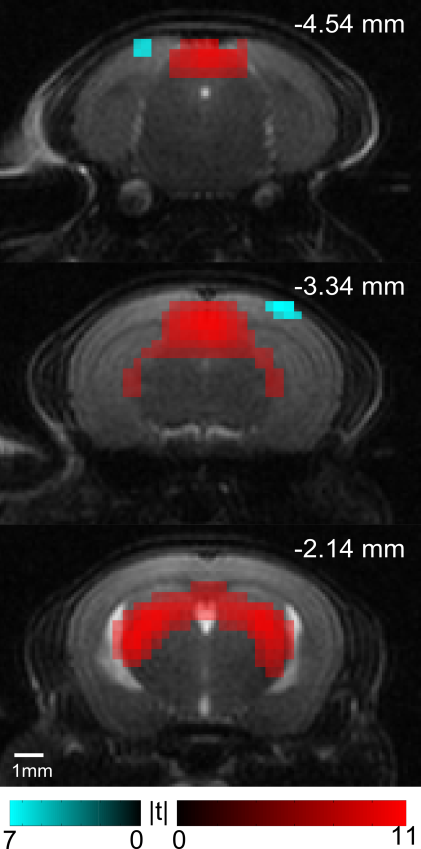


Figure S1. Mixed effects multi-subject analysis (two-tailed t-test, FWER p < 0.05 (T > ~4.28 for positive BOLD responses (red) and T > ~4.34 for negative BOLD responses (blue), determined through permutation testing)). Three coronal slices with distance from Bregma are shown.
